# Electrical Resistivity of 3D-Printed Polymer Elements

**DOI:** 10.3390/polym15142988

**Published:** 2023-07-08

**Authors:** Stanislav Stankevich, Jevgenijs Sevcenko, Olga Bulderberga, Aleksandrs Dutovs, Donat Erts, Maksims Piskunovs, Valerijs Ivanovs, Victor Ivanov, Andrey Aniskevich

**Affiliations:** 1 Institute for Mechanics of Materials, University of Latvia, Jelgavas St. 3, LV-1004 Riga, Latviaolga.bulderberga@lu.lv (O.B.); andrejs.aniskevics@lu.lv (A.A.); 2 Institute of Chemical Physics, University of Latvia, Jelgavas St. 1, LV-1004 Riga, Latvia; 3 ZRF Ritec SIA, Gustava Zemgala St. 71A, LV-1039 Riga, Latviav.ivanov@ritec.lv (V.I.)

**Keywords:** fused filament fabrication, electrical resistivity, structural levels, traxel, monolayer, anisotropy, multilayer structure, annealing, Joule heating, piezoresistivity

## Abstract

During this study, the resistivity of electrically conductive structures 3D-printed via fused filament fabrication (FFF) was investigated. Electrical resistivity characterisation was performed on various structural levels of the whole 3D-printed body, starting from the single traxel (3D-printed single track element), continuing with monolayer and multilayer formation, finalising with hybrid structures of a basic nonconductive polymer and an electrically conductive one. Two commercial conductive materials were studied: Proto-Pasta and Koltron G1. It was determined that the geometry and resistivity of a single traxel influenced the resistivity of all subsequent structural elements of the printed body and affected its electrical anisotropy. In addition, the results showed that thermal postprocessing (annealing) affected the resistivity of a standalone extruded fibre (extruded filament through a printer nozzle in freefall) and traxel. The effect of Joule heating and piezoresistive properties of hybrid structures with imprinted conductive elements made from Koltron G1 were investigated. Results revealed good thermal stability within 70 °C and considerable piezoresistive response with a gauge factor of 15–25 at both low 0.1% and medium 1.5% elongations, indicating the potential of such structures for use as a heat element and strain gauge sensor in applications involving stiff materials and low elongations.

## 1. Introduction

The use of additive manufacturing combined with other innovative technologies of Industry 4.0 allows for the quick and precise development of a product with complex geometry and properties. The popularity of using FFF technology arises not only for personal purposes but also in mass production. In addition, due to the flexibility of FFF, the printing process can be carried out using a wide range of different materials [1], which allows for the creation of parts with completely different physical properties. As a result, FFF product manufacturing is increasingly being introduced into various industry sectors, such as aerospace, medical devices and dentistry, mechanical engineering, automotive, etc. [2].

Fused filament fabrication technology approbated for printers with two or more nozzles has made it possible to create complex structures by combining multiple materials [3]. One of the options for such technology is the creation of special purpose printed products with embedded electrically conductive elements using conductive filament [4,5]. In this way, printed objects can be afforded the properties of electromagnetic and radio frequency shielding, 2D and 3D circuits, various sensors, de-icing, and damage detection [6,7,8]. Special or multipurpose advanced 3D-printed parts, depending on their application, require specific electrical parameters, which should be estimated before manufacturing.

The literature data showed that the internal structure (printing orientation and pattern) of printed products strongly influences their electrical resistivity [9,10,11,12,13]. The influence of the structure of a printed product on its resistivity is explained by the presence of contact resistance between traxels, which form the entire product [14,15,16]. Thus, the resistivity of the printed product varies based on the number of traxels, their shapes, and their arrangement within the whole printed body. However, preliminary data suggest that the factor of contact resistance between traxels is not the only significant factor affecting the resistivity of a printed product. There is a basis to support the statement that the electrical characteristics of an initial filament could undergo modification during the extrusion process through a printer nozzle, which could be described by the structural change. Many studies have investigated the structure and property change in the material after printing, where the effects of shape memory [17] and increased crystallinity [18,19] took place. If such effects cause a change in the electrical resistivity of a filament after printing, it could mean that the overall pattern of electrical resistivity of a printed part should be determined by the electrical resistivity of a traxel, rather than an initial filament. One of the crucial purposes of this work was to show the presence of change in electrical properties during transitions between structural levels of a printed part. The aim of this study was to reveal the basic principles of structure formation of 3D-printed conductive parts and their electrical properties and to prove the possible use of the parts as electrically active elements. In the given context, the research tasks were formulated as follows: (1) identification of the contact resistance influence towards electrical resistivity measuring of thermoplastic material with conductive nanofillers; (2) characterisation of electrical conductivity of the 3D-printed structure starting from elementary traxel; monolayer and multilayer structures; and hybrid structures of basic nonconductive polymers and electrically conductive ones. As a concluding step, (3) the thermoelectrical and piezoresistive properties of imprinted active elements for applications should be revealed.

Investigating the resistivity of a traxel and following structural levels was necessary to shed light on the overall picture of the resistivity nature of printed multilayer structures. The results can help to identify the influencing factors on the resistivity of a printed part, thus assisting its electrical performance optimisation.

## 2. Materials and Methods

### 2.1. Materials

Conductive polylactic acid (PLA) filament Proto-Pasta supplied by ProtoPlant INC (Vancouver, WA, USA) and conductive polyvinylidene fluoride Koltron G1 filament supplied by ADDNORTH (Stockholm, Sweden) with a diameter of 2.85 mm were utilised. The additives responsible for the conductivity of Proto-Pasta and Koltron G1 filaments were carbon black and Aros graphene^®^, respectively. For specimens with embedded conductive elements, the following materials were used: Koltron G1 was used for the conductive elements; Tough PLA White supplied by Ultimaker was used as a nonconductive base. Electrical contacts on the surface of specimens were made using silver conductive paint (SCP) ELECTRON 40 AC (Amepox Microelectronics, Lodz, Poland).

### 2.2. Testing Methods and Printing Parameters

**Figure 1:**
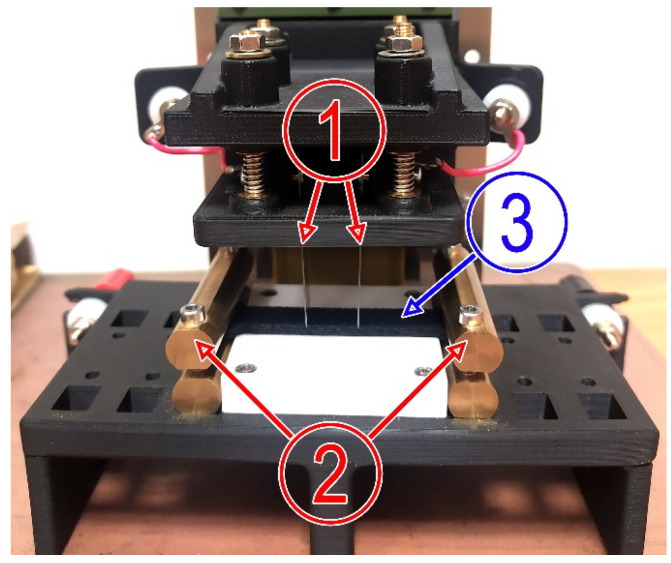
Four-point probe resistivity measuring tool with voltage (1) and current (2) probes and specimen locked inside (3).

Specimens used in this study were produced using a two-nozzle printer—Ultimaker S5 (Utrecht, The Netherlands). Model slicing, setting up printing parameters and other preprint finalising was carried out using Ultimaker Cura software. Electrical resistivity measurements using the two-probe determination method [20] were performed using Multimeter Tektronix DMM 4020 (Tektronix, Beaverton, Oregon, USA). For approbation of the four-probe determination method [20], a special measurement tool (Figure 1) designed at ZRF Ritec SIA (Riga, Latvia) was used. The device was specially constructed to measure the resistivity of 3D-printed thermoplastic specimens. Outer current probes (2) (Figure 1) strongly held a specimen in one place, while inner voltage probes (1) were pressed towards specimen (3) till the constant electrical signal was detected.

Tensile tests were executed according to ISO 527-1 standard using the universal testing machine Zwick 2.5k (Zwick Roell Group, Ulm, Germany). Displacement measurements were performed using clip-on extensometer TC-EXICLEL. Optical and scanning electron microscope (SEM) images were obtained using OLYMPUS bx51 and Hitachi FE-SEM s4800 microscopes (Tokyo, Japan), respectively. Thermal imaging of specimens during electrical tests was performed using RS PRO RS-9875 thermal imaging camera (RS Components, Corby, United Kingdom). Tests within this research were performed at room temperatures of 21–22 °C and at 35–40% humidity.

Joule heating and piezoresistivity tests were conducted on hybrid dog-bone specimens (prepared according to ISO 527-2 standard) with gauge section dimensions of 75 × 10 × 3 mm. Hybrid specimens were made from two materials: the nonconductive base was made from Tough PLA White (Ultimaker), and the longitudinally imprinted conductive element with dimensions of 80 × 6 × 1 mm was made from Koltron G1. Electrodes were fused to the ends of the conductive part of the specimen and covered with SCP.

The main printing parameters used in this study were chosen according to recommendations of the filaments’ suppliers to achieve better quality of printed parts. Set printing parameters are summarised in Table 1. A printer nozzle with a diameter of 0.4 mm was used unless otherwise specified.

#### Electrical Resistivity Determination Methods

**Figure 2:**
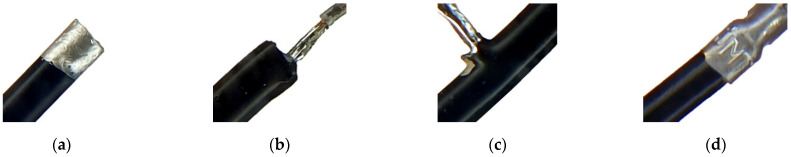
Electrical contact types for resistivity measuring of the filament: coated contacts using SCP (**a**), contacts with 0° (**b**) and 90° (**c**) fused electrodes, and crimped contacts (**d**).

**Figure 3:**
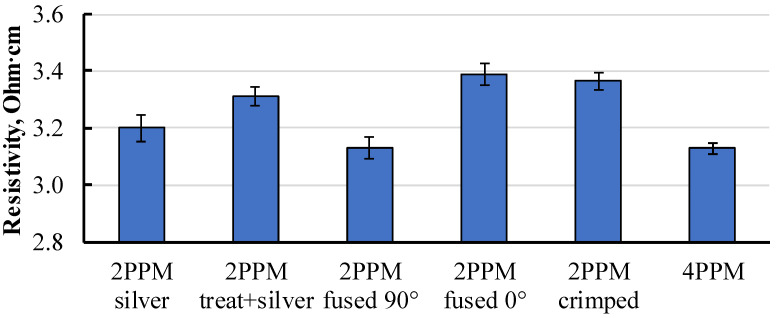
Filament resistivity obtained using various methods.

During determination of the electrical resistivity of nanomodified thermoplastic specimens, contact resistance between the electrode and the specimen should be considered. To assess the effect of contact resistance, several measurement methods were compared. Resistivity testing methods were approbated directly on the filament to avoid the appearance of additional resistance introduced by the specificity of the printed structure. Specimens were prepared from Proto-Pasta filament. Six measuring methods were approbated and compared: 2-point probe method (2PP) with coated contacts using SCP (Figure 2a); 2PP with prior surface treatment (using 800–1200 grit sandpaper) and coated contacts using SCP; 2PP with parallel and transversal fused metal contacts (Figure 2b,c) relative to extrusion direction of the filament; 2PP method with crimped contacts (Figure 2d); 4-point probe method (4PP) using special resistivity measuring tool (Figure 1) and SCP-coated contacts. The resistivity data acquired using the mentioned methods are presented in Figure 3.

Specimens measured using the two-point probe method with perpendicularly fused electrodes and the four-point probe method with coated SCP showed the lowest resistivity at 3.13 ± 0.05 Ohm·cm.

The data indicated the presence of contact resistance, which may increase the measurement error. Such a problem was encountered in previous studies [9,13,21], and the most effective way for the 2PP method to minimise the effect of contact resistance was to use an SCP as a connection medium between the material and probe. The other option was to use the 4PP method. However, it is not always practical to use the four-point method for determining the resistivity in the case of a small-sized specimen or when inner voltage probes can influence the measurement process. For instance, during thermal imaging, the voltage probes can act as heat dissipation elements, therefore affecting the heat distribution map. According to results obtained during this study, an alternative to the 4PP method could be the 2PP method with fused electrodes perpendicular to the extrusion (or perpendicular to the direction of printing in the case of printed parts).

## 3. Results and Discussion

### 3.1. Electrical Resistivity of Structural Levels

To assess the factors that influence the resistivity of a 3D-printed final part, its structure was segmented into distinct levels: traxel, monolayer, and multilayer structure. The electrical resistivity was investigated on each structural level.

#### 3.1.1. Traxel

**Figure 4:**
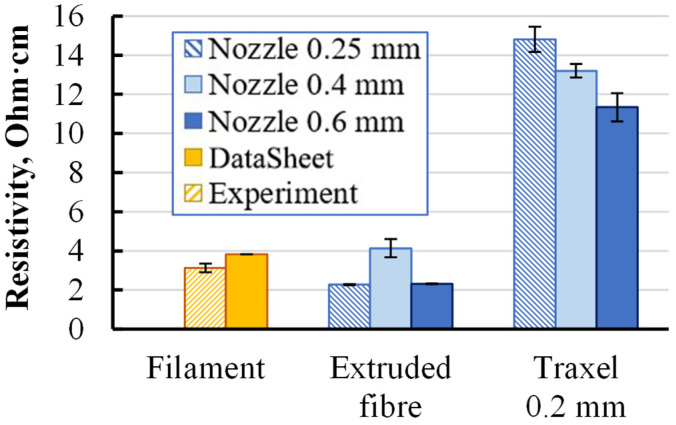
Resistivity data of Proto-Pasta PLA in various states.

To characterise the electrical resistivity of the smallest structural unit, represented as a traxel, the filament was investigated before and after extruding through the printer nozzle. Specimens were divided into the following groups: source filament, extruded fibre, and traxel. The fibre was extruded by the printer nozzle in freefall without a printing bed. The traxel was the single fibre extruded by a nozzle and printed separately on the printer bed in a single pass. The layer thickness of the traxel was 0.2 mm. The resistivity of each specimen group printed with nozzle diameters of 0.25, 0.4, and 0.6 mm was measured and presented in Figure 4. A significant increase in resistivity of 3–4 times for traxels compared with extruded fibre and filaments was observed for all nozzle diameters.

**Figure 5:**
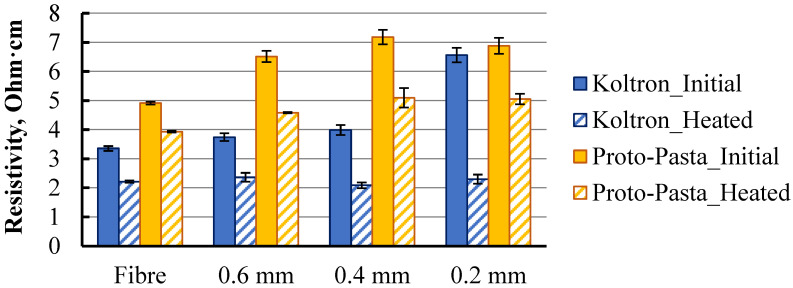
Resistivity data of annealed and nonannealed Koltron G1 and Proto-Pasta extruded fibres and traxels with various thicknesses produced by a nozzle with a diameter of 0.4 mm.

Such a high difference in resistivity between extruded fibre and traxel indicated the presence of structural changes in the material during the printing procedure. For additional data, the resistivity of Koltron G1 filament specimens was measured. Specimens made from Proto-Pasta coil supplied two years apart from the previously tested one were also re-examined. The reason for the re-examination was the resistivity of the new coil filament, which was 5.1 Ohm·cm (the previous one was 3.1 Ohm·cm). Several groups of extruded fibres and traxels with different layer thicknesses were tested and the results are presented in Figure 5.

**Figure 6:**
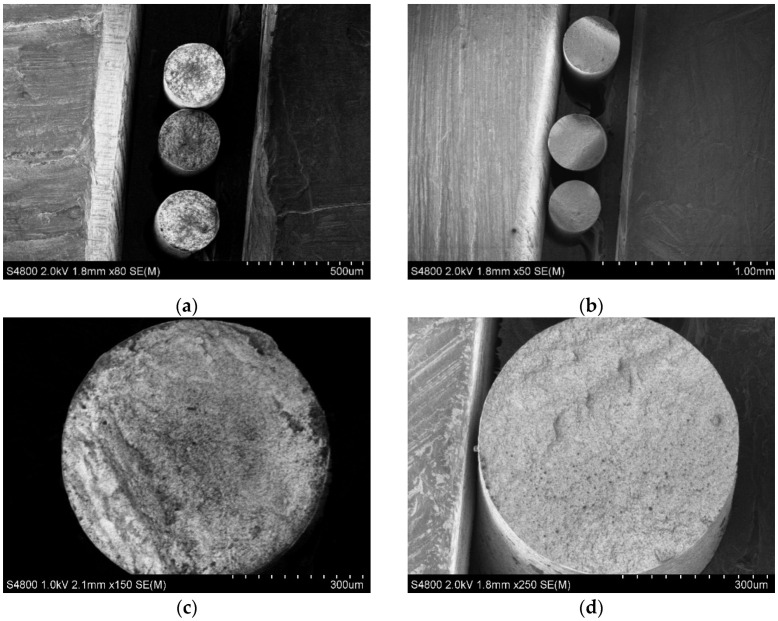
SEM images of Proto-Pasta extruded fibres with nozzles Ø 0.25 (**a**,**b**) and 0.4 mm (**c**,**d**) before (**a**,**c**) and after (**b**,**d**) thermal postprocessing.

**Figure 7:**
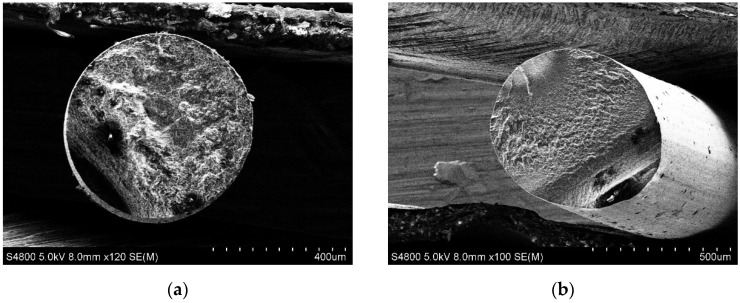
SEM images of Koltron G1 extruded fibres before (**a**) and after (**b**) thermal postprocessing.

The trend of resistivity increasing with traxel formation, as well as subsequent thinning of the specimen, was observed, as in the previous result (Figure 4). The relationship between the specimen’s cross-section geometry and its resistivity may indicate the conductivity heterogeneity of the specimen along its cross-section. Such heterogeneity was considered as multiple zones with different conductivity values distributed over a cross-section of extruded fibre or traxel. Visual inspection was conducted on images obtained using SEM (Figure 6a,c and Figure 7a). In this case, the property of SEM to transform the obtained electron signal from the surface of the specimen into a 2D intensity map was used. A pixel’s brightness on the obtained image is proportional to the signal intensity captured using the detector at the respective point on the specimen’s surface. Thus, based on the nonuniform intensity of the obtained image within the cross-section of a single specimen, one can judge the presence of zones with different electrical properties [22].

To achieve low surface roughness of the studied specimens’ cross-sections, they were intentionally fractured in a brittle manner. Considering the amorphous and semicrystalline nature of the inspected materials [23], to achieve brittle fracturing, specimens were precooled by holding them in liquid nitrogen for several seconds. This made it possible to obtain a cut without zones of viscous fracture, thereby providing a good surface for study using SEM. In the obtained images, zones of different brightness were visible on the cross-section of Proto-Pasta extruded fibres produced using nozzles with diameters of 0.25 and 0.4 mm (Figure 6a,c). Two distinct zones were more pronounced: one located at the centre of each specimen and the other nearer to the edges. Given that the specimens were conductive and not additionally coated, the obtained result was interpreted as the presence of two zones inside the specimens with different electrical properties. The mentioned zones could hypothetically explain the change in resistivity of the filament during traxel formation and its thinning. With one zone located near the walls and the other in the central part of the printed element, the areas of such zones may vary during the formation of traxels of different thicknesses. Therefore, the overall resistivity of the printed element, which is the result of the combination of two or more zones, may vary depending on the thickness of the element. The formation of these zones can be justified by the uneven nanofiller distribution due to the stresses created by the nozzle during application of molten material onto the substrate or previous layer, the sharp temperature gradient of the material as it is extruded from a heated nozzle to the open area, and the temperature change during the distribution of molten material onto a base with a temperature significantly lower than the melting temperature of the material.

The smooth cross-sectional surface of Koltron G1 specimens was not achieved even with brittle fracture. Several specimen groups produced with different nozzles were used. In all cases, surface roughness was significant, hindering successful SEM analysis. Different zones in the Koltron G1 specimens could not be distinguished (Figure 7a). Thus, it was impossible to determine whether it was the absence of zones or the inapplicability of the methodology for their detection in the inspected material.

Diverging results obtained through visual analysis using SEM and the irregular relationship between traxels’ thicknesses and their resistivity (Figure 4 and Figure 5) impedes the quantitative determination of different zones within one element. Therefore, additional studies, including multiple groups of specimens printed with different printing parameters (nozzle diameter, layer thickness, printing temperature and speed, etc.), are required to investigate the nature and factors affecting the resistivity behaviour at the smallest printed structural element. The task of this study was to highlight the presence of this phenomenon.

#### 3.1.2. Annealing

Thermal treatment was utilised in various studies aimed at enhancing the properties of printed parts [24,25,26,27,28]. In these studies, the post-annealing enhancement in properties was attributed to the strengthening of the bonds between the traxels. Verification was conducted during this study to substantiate the presence of structural changes even within a single traxel during thermal treatment. Thus, electrical property changes within a single traxel could be considered as an additional factor impacting the electrical properties of printed multilayer structures, in addition to the influence of already discovered contacts between traxels [14,15,16].

Extruded fibres and traxels of Koltron G1 and Proto-Pasta were subjected to annealing, similar to [24,25,26]. In the mentioned studies, the highest effect of annealing on the mechanical properties of conductive PLA was obtained at the temperature of 30 °C above the glass transition temperature. Considering the glass transition temperature of Proto-Pasta conductive PLA, 60–65 °C, the temperature for its annealing during this study was chosen as 100–110 °C. In the absence of specific annealing time through various studies, an average of 10 min was taken. According to the Koltron G1 datasheet, the material’s glass transition temperature is −34 °C and it has high thermal resistance up to 110–120 °C, up to which the material experiences low thermal molecular mobility. To surpass this threshold, annealing of Koltron G1 was performed at 120–130 °C for 10 min.

Specimens that underwent annealing were subjected to resistivity tests and visual analysis using SEM imaging. Obtained resistivity data are presented in Figure 5 and SEM images in Figure 6b,d and Figure 7b. The resistivity of all specimen groups, both Proto-Pasta and Koltron G1, decreased. Furthermore, the resistivity values of all Koltron specimens reached a fixed minimum of 2.1–2.3 Ohm·cm. Based on the annealed Koltron specimens, no dependence of resistivity on geometry could be observed anymore. The obtained results indicated changes that occurred within the specimens due to annealing, as evidenced not only by the resistivity data but also by several SEM images (Figure 6b,d). The presented SEM images of Proto-Pasta specimens demonstrated the disappearance of areas with different brightnesses within the specimen’s cross-sections. The cross-sections had a more uniform intensity distribution than before annealing. Thus, it can be assumed that the annealing process contributed to a change in the structure of extruded fibres. An alternative explanation for the brightness change in the acquired post-annealed images could be the reduction in the specimen’s surface roughness, as was proven in [26]. However, nothing had changed visually for the Koltron G1 specimens, since no systematic structure was detected even before annealing.

The obtained results indicated the relationship between the thickness of the printed traxel and its resistivity. In addition, the annealing procedure permanently affected the resistivity of extruded fibres and traxels. It was proven that morphological changes could occur during printing and thermal postprocessing even within a single elementary printing unit-traxel. Therefore, this factor remains overlooked in many existing studies and should be examined more closely.

#### 3.1.3. Anisotropic Monolayer

**Figure 8:**
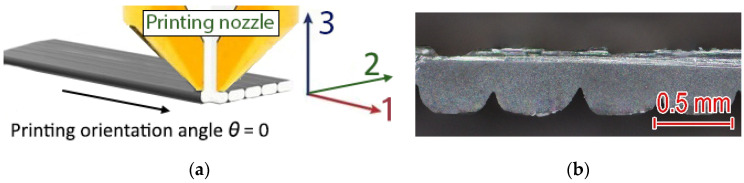
Scheme of (**a**) 3D-printed monolayer and its cross-section (**b**) obtained with an optical microscope.

Within the scope of this study, the monolayer was the subsequent structural printing element following the traxel. A monolayer was considered a two-dimensional structure comprising a set of parallel traxels aligned with axis 1 (Figure 8) [25].

The electrical conductivity of such a system as a second-rank tensor could be defined with only two independent components along the main axes of symmetry, i.e., axes 1 and 2.

(1) $$\sigma_{ij}=\left(\begin{array}{ccc} \sigma_{11} & 0 & 0\\ 0 & \sigma_{22} & 0\\ 0 & 0 & 0 \end{array}\right)$$

where *σ*_11_ is specimen conductivity along the printing direction and *σ*_22_ is specimen conductivity transversal to the printing direction in the 1–2 plane. The conductivity of the specimen in the specific case of printing direction angle *θ* rather than 0° was calculated using the components *σ*_11_ and *σ*_22_, similar to the previous study [29], where such calculations were performed for a unidirectionally fibre-reinforced composite:

(2) $${\sigma^{\prime }}_{11}=\sigma_{11}\cos^{2}\theta +\sigma_{22}\sin^{2}\theta \;$$

where *σ′*_11_ is specimen conductivity at a specific printing angle *θ*. Components *σ*_11_ and *σ*_22_ were experimentally predetermined. Conductivity data were converted using (3) and presented in the form of electrical resistivity to unify with other data presented within the scope of this study:

(3) $${\rho^{’}}_{11}=\frac{1}{\frac{1}{\rho_{11}}\cos^{2}\theta +\frac{1}{\rho_{22}}\sin^{2}\theta \;}$$

**Figure 9:**
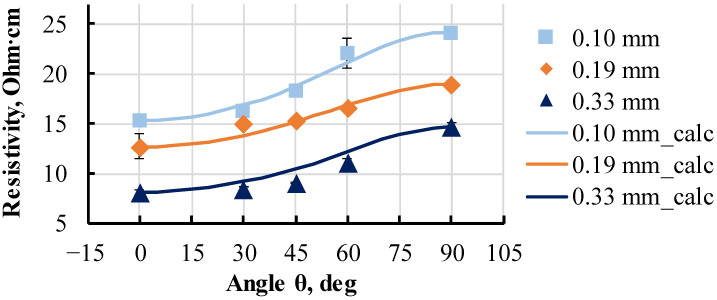
Calculated (3) and experimental resistivity of monolayer Proto-Pasta specimens with various thicknesses and printing orientation angle *θ*.

Several groups of 120 × 10 mm Proto-Pasta PLA monolayer specimens printed with a Ø 0.4 mm nozzle; layer thicknesses of 0.10, 0.19, and 0.33 mm; and printing orientations of 0, 30, 45, 60, and 90° were investigated. Calculated using (3) and experimental resistivity data were obtained for all groups and are presented in Figure 9. The cross-section area of specimens was determined via image analysis of obtained images under an optical microscope.

All groups had a similar degree of anisotropy up to two times, caused mainly by the contact resistance between traxels and potentially by the irregular distribution of resistive zones in a specimen’s cross-section, as described earlier. For specimen groups at printing orientation of 0° and thicknesses 0.10, 0.19, and 0.33 mm, the resistivity values were 15 ± 0.5, 13 ± 1, and 8.2 ± 0.2 Ohm·cm, respectively. The thinner the specimens were, the higher their resistivity was. The trend of resistivity reduction with increasing specimen thickness corresponded to the results obtained for individual traxels (Figure 4 and Figure 5). The calculated data aligned with the trend of increased resistivity with a printing angle deviating from 0°. Experimental and calculated values of the group with thicknesses of 0.10 and 0.19 mm were in good agreement. The resistivity of specimens with a thickness of 0.33 mm only varied within a 1% range with an increase in printing angle from 0 to 45°. For angles more than 45°, the resistance increased more rapidly. Such nonuniform dependence was not accurately described using the calculated data.

The obtained results for the monolayer specimens aligned well with the results obtained for traxels, where the same thickness-dependent resistivity trend was observed (Figure 5). Therefore, this proved that the geometry of the single traxel might influence not only its resistivity but also the resistivity of all subsequent structural elements of the printed part.

#### 3.1.4. Multilayer Structure

**Figure 10:**
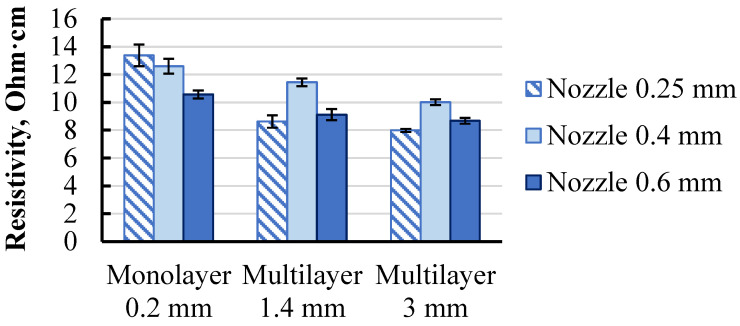
Resistivity data of Proto-Pasta conductive PLA monolayer and multilayer structures with a printing layer thicknesses of 0.2 mm.

Followed structural elements from monolayer to multilayer were investigated. The resistivity of specimens with a constant layer thickness of 0.2 mm and printed with nozzle diameters of 0.25, 0.4, and 0.6 mm was measured and presented in Figure 10. The multilayer structure was represented by two groups of specimens with thicknesses of 1.4 and 3 mm, aiming to observe a potential dependence of resistivity on specimen thickness.

Comparing a monolayer with a multilayer structure, the resistivity dropped by 10–30% for all specimen groups. Such behaviour could be described by the specificity of the printing procedure. During the printing of the second and following layers of a multilayer structure, the nozzle heats the previous layer, thus creating a better connection between the printing traxel and the previous layer, potentially creating a more united structure between two adjacent traxels in the z-axis, thus providing thicker conductive media. This nicely aligns with the previous results, which showed that as the traxel thickened, its resistivity decreased (Figure 5). The subsequent thickening of the multilayer specimens from 1.4 to 3.0 mm had little effect on their resistivity.

**Figure 11:**
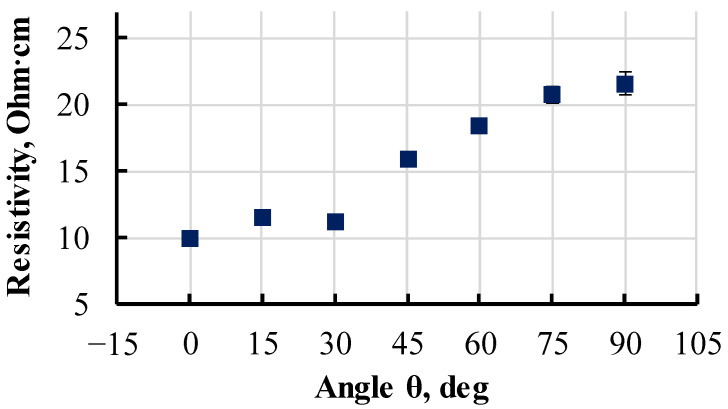
Resistivity data of multilayer Proto-Pasta specimens with various printing orientation angles *θ*.

The group of five multilayer specimens with dimensions of 50 × 10 × 3 mm and printing orientations of 0, 15, 30, 45, 60, 75 and 90° was investigated. Specimens were printed with a Ø 0.4 mm nozzle and layer thickness of 0.2 mm. Averaged resistivity data for all specimens are presented in Figure 11.

All specimens showed great similarity, so error bars were barely visible. Multilayer specimens had a similar degree of anisotropy to that of the monolayers of up to two times, caused by the contact resistance between traxels and potentially by the irregular distribution of the specimen’s resistivity, as described earlier.

The obtained resistivity results at all structural levels demonstrated that the formation of the traxel during printing led to a noticeable increase in resistivity compared to the initial filament. Moreover, by transitioning to subsequent structural levels, the resistivity decreased. This demonstrated the significance of investigating the electrical properties of printed parts at individual structural levels.

### 3.2. Active Conductive Elements

The previous sections discussed the factors of printing and the material’s structure parameters that affected the electrical resistivity of printed parts. In this chapter, the thermal and mechanical influence factors were investigated as the most encountered in real industrial applications [12,30,31,32].

#### 3.2.1. Temperature Sensing

**Figure 12:**
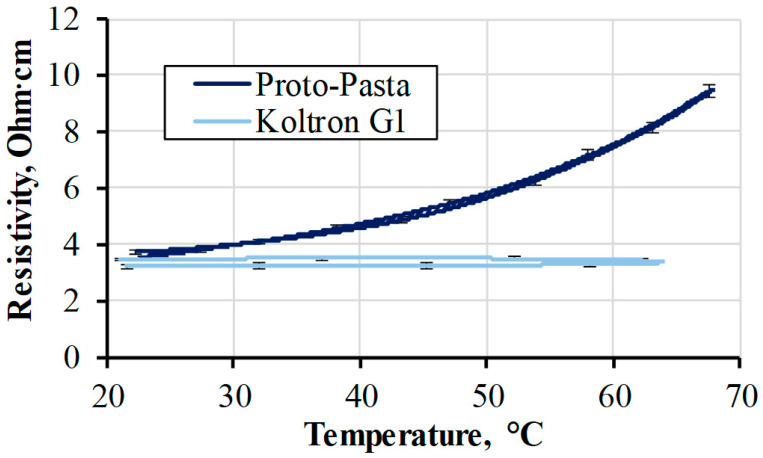
Proto-Pasta and Koltron G1 filaments’ resistivities during the full heating cycle.

One of the crucial environmental factors affecting the electrical properties of printed parts is their temperature. The resistivity of conductive PLA is known to vary with temperature change even in the diapason of 25–50 °C [33]. The temperature–resistivity relationship was obtained for Koltron G1 and Proto-Pasta filament specimens with fused electrodes. The temperature range for testing was chosen in the margins of the examined materials’ softening temperature [34], beyond which the material is not likely to be used in real applications as a sensor. The averaged resistivity curves obtained from several specimens for each material were demonstrated in Figure 12.

The measurements were taken each 20 s, while the temperature increased to 65 °C and cooling with a heating rate of 0.1 °C/min. During heating, a resistivity increase of 150% was noticed for Proto-Pasta. However, Koltron G1 showed no resistivity change whatsoever. The resistivities of both filaments returned to their original values after cooling. Koltron G1 showed good thermal stability within the range of 65 °C. The resistivity of Proto-Pasta, on the other hand, even near room temperatures, was still temperature-dependent. Results showed how distinctive resistivity responses of two different filaments could be during heating, despite their identical initial resistivity at room temperature. Considering the obtained resistivity data, Koltron G1 can be utilised in applications that require stable resistivity values when the material is operated within the range of 70 °C. Proto-Pasta, in turn, demonstrated a responsiveness of resistivity to its temperature. In comparison, the resistance of the commonly used platinum resistance temperature sensor PT100 only varies by 15% when the temperature rises from 20 to 70 °C [35]. Therefore, the active printed element made from Proto-Pasta has the potential to be used as a temperature sensor.

#### 3.2.2. Joule Heating

**Figure 13:**
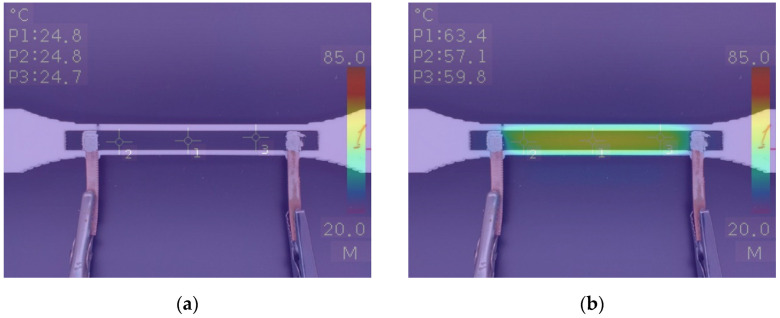
Combined regular and thermal images of a 3D-printed dog bone with a conductive part at the initial stage (**a**) and under an applied voltage of 20 V (**b**). Values P1, P2, and P3 represent the temperature at respective points P1, P2, and P3 at the surface of the specimen.

The conductive part of the hybrid specimen was subjected to voltages of 5, 10, 15, and 20 V to obtain time–temperature dependences. Thermal imaging analysis was conducted during electrical tests. Combined optical and thermal images are presented in Figure 13.

**Figure 14:**
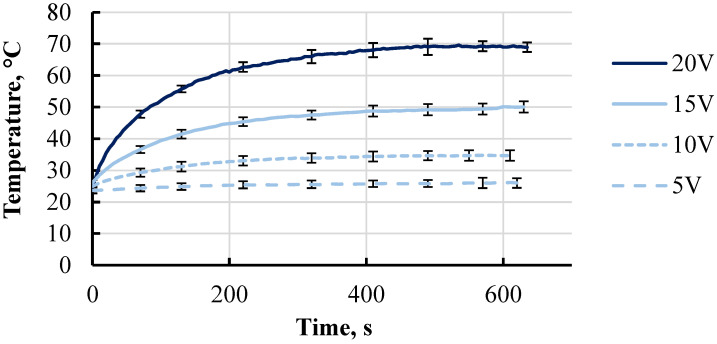
Time–temperature curves of printed conductive elements at 5, 10, 15, and 20 V voltages.

The voltage was applied until the temperature of the inspected conductive part reached a plateau. Thermographic analysis of the specimen showed that the heat distribution was homogeneous along the whole conductive part, except the area near the electrodes. Heat in those areas was dissipated due to the fused copper electrodes. Areas exhibiting more pronounced temperature gradients were observed only at 20 V and 65 °C. Temperature data were collected every 10 s from the central point P1 of the specimens during the heating process. The averaged temperature curves for each group of three tested specimens are shown in Figure 14. As expected, when a voltage was applied to the specimens, the temperature started to increase. The curves obtained under the different voltages showed that the temperature increased more dynamically with a higher voltage.

In 600 s, the average temperature increased up to 26, 35, 50, and 65 °C at 5, 10, 15, and 20 V, respectively. Even at a voltage of 10 V, a temperature of 30 °C was already achievable under 100 s. It showed that the material still could be used as a heating element even within applications where high voltages are not easily achievable due to limited power or other circumstances.

**Figure 15:**
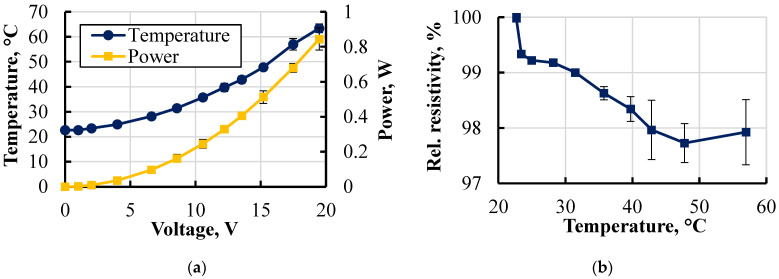
Graphical representation of the voltage–temperature/power relationships (**a**) and temperature-relative resistivity dependence (**b**).

The power necessary to maintain the heating element at a certain temperature was calculated as *P* = *I × U*. In those tests, temperature and resistance data were collected in their equilibrium state at each voltage increment step from 1 to 20 V (Figure 15).

At each respective voltage step, the power was calculated and is presented in Figure 15a. A power of 0.9 W was achieved at 20 V. Along with that, specimens exhibited a decrease in resistivity by 2–3%, demonstrating behaviour closely resembling that of the Koltron G1 filament used for conductive track printing (Figure 12). The obtained result indicated the thermal stability of the resistivity of the printed conductive element. Hence, this provided insight into the predictable thermoelectrical behaviour of embedded printed parts used as heating elements. To further assess long-term thermal stability, it is necessary to conduct additional experiments involving multiple heating cycles for supplementary verification.

#### 3.2.3. Piezoresistivity

**Figure 16:**
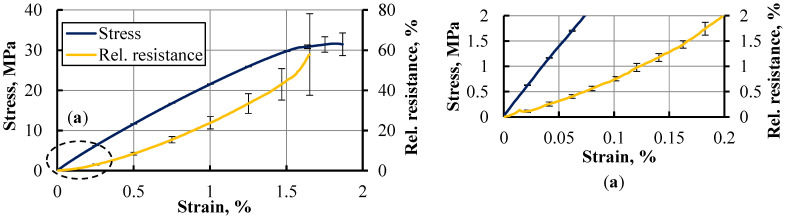
Relative resistance of the imprinted conductive elements and stress as functions of strain and the initial segments of the curves (inset **a**).

The electromechanical performance of 3D-printed hybrid dog bone specimens with imprinted active elements was carried out by measuring the resistance under longitudinal tension at 1 mm/min. Resistance readings were taken at a frequency of 5 Hz. Each experiment was conducted until either the specimen was fractured or the signal was lost. Four hybrid specimens from distinct printing sessions were taken. The stress–strain curves of individual specimens showed very high similarity. Error bars of the averaged stress–strain curve are barely visible in Figure 15. The results exhibited a relationship between relative resistance and tensile strain (Figure 16), with resistance gradually rising as the strain increased, signifying a pronounced electromechanical response. The highest level of strain, ranging from 1.5 to 1.7%, was attained during tensile testing, with a corresponding increase in resistance up to 60%. Tested specimens showed a high level of similarity in the resistance–strain relationship, within a range of 0.025–1% of strain, indicating excellent repeatability of piezoresistive response among separately printed specimens. Even at the small strain of 0.2% or lower, the resistance response remained distinctly perceptible.

The sensitivity of the conductive elements was expressed by a gauge factor GF, calculated using (4).

(4) GF=ΔRR0ΔLL0

where Δ*R*, Δ*L*, *R*_0_, and *L*_0_ represent resistance change under strain, elongation, initial resistance, and initial length, respectively. A gauge factor of 16 was obtained for a strain of 0.5%, which increased to 22 as the strain raised to 1%. Results revealed a considerable electromechanical response at both medium and low levels of elongation, coupled with good reproducibility between specimens derived from distinct printing sessions, therefore indicating its potential for use as a strain gauge sensor in applications involving stiff materials and low elongations.

## 4. Conclusions

During this study, the electrical resistivity of the 3D-printed conductive parts was investigated at various structural levels. The resistivity of the multilayered structure was investigated under variable mechanical and temperature conditions.

Two methods, 4PP and 2PP, with transversally fused electrodes, were identified as the most accurate methods for determining the electrical resistivity of conductive PLA filled with carbon black. Thus, 2PP can be used as an alternative to the 4PP method in cases where the latter cannot be applied.

Resistivity was examined at different structural levels of 3D-printed parts, including traxel, monolayer, and multilayer structures. It was found that the resistivity significantly increased during the transition from extruded fibre to traxels but decreased with further structural levels. In addition, the cross-sectional geometry of traxels showed an influence towards their resistivity. The thickness reduction in the traxel printed from both Proto-Pasta and Koltron G1 increased its resistivity. Such behaviour was justified by the heterogeneity of conductivity across the cross-section of the specimen. The heterogeneity was considered as multiple zones with different conductivity values distributed over a cross-section of extruded fibre or traxel. Both groups of traxels and extruded fibres from Proto-Pasta and Koltron G1 exhibited a decrease in resistivity after annealing. Obtained results indicated that structural changes could occur within a single traxel not only by its thickness variation but also during thermal postprocessing. The identification of those factors provides further insights into the nature of electrical conductivity in FFF-printed objects. This can serve as a basis for developing new electrical conductivity models for printed objects and for advancing thermal postprocessing technologies.

The influence of printing angular orientation on printed monolayers’ resistivity was investigated. It was observed that the resistivity along the printing orientation was approximately 30–40% lower than in the transversal direction. The reason for this was caused by the contact resistance between traxels and potentially by the irregular distribution of resistive zones in a specimen’s cross-section. As a result, working with complex 3D structures or imprinted parts using conductive materials, it is necessary to consider the printing direction to maintain the constant resistivity of the printed element.

The resistivity reduction with the increase in the monolayer’s thickness showed similar behaviour in comparison to the results obtained for individual traxels. Therefore, the geometry of the single traxel may influence not only its resistivity but also the resistivity of all subsequent structural elements of the printed part.

The thermoelectrical and piezoresistive properties of 3D-printed active elements were studied. Proto-Pasta demonstrated a responsiveness of resistivity to its temperature, showing the potential of using such material for imprinted temperature sensors. Koltron G1, in turn, exhibited good thermal stability within the range of 70 °C, demonstrating the possibility of using it in printed conductive structures as a heating element. In addition, Koltron G1 showed a considerable electromechanical response at both medium and low levels of elongation with a gauge factor of 15–25, which indicated its potential for use as a strain gauge sensor in applications involving stiff materials and low elongations.

The knowledge gained from this study provides insight into the nature of resistivity of multilayer structures. These results can help to identify the influencing factors on the printed part’s resistivity, thus assisting in the electrical performance optimisation of printed conductive parts and contributing to advancement in the field of printed electronics.

## Figures and Tables

**Table 1:** Printing parameters.

	Filaments		
Parameters	Proto-Pasta	Koltron G1	Tough PLA White
Printing speed (mm/s)	30	20	30
Printing temperature (°C)	215	265	215
Substrate temperature (°C)	60	60	60
Material infill (%)	100	100–105	100

## Data Availability

Available in ZENODO repository: https://zenodo.org/record/8093932.
